# Inactivity of Stat3 in sensory and non-sensory cells of the mature cochlea

**DOI:** 10.3389/fnmol.2024.1455136

**Published:** 2024-10-14

**Authors:** L. Bieniussa, C. Stolte, P. Arampatzi, J. Engert, J. Völker, R. Hagen, S. Hackenberg, K. Rak

**Affiliations:** ^1^Department of Oto-Rhino-Laryngology, University Hospital, Würzburg, Germany; ^2^Core Unit System Medicine, University of Würzburg, Würzburg, Germany

**Keywords:** Stat3, cochlea, outer hair cells, supporting cells, organ of Corti, inflammation, hearing impairment

## Abstract

Signal transducer and activator of transcription 3 (Stat3) plays a role in various cellular processes such as differentiation, inflammation, cell survival and microtubule dynamics, depending on the cell type and the activated signaling pathway. Stat3 is highly expressed in the hair cells and supporting cells of the cochlea and is essential for the differentiation of mouse hair cells in the early embryonic stage. However, it is unclear how Stat3 contributes to the correct function of cells in the organ of Corti postnatally. To investigate this, an inducible Cre/loxp system was used to knock out Stat3 in either the outer hair cells or the supporting cells. The results showed that the absence of Stat3 in either the outer hair cells or the supporting cells resulted in hearing loss without altering the morphology of the organ of Corti. Molecular analysis of the outer hair cells revealed an inflammatory process with increased cytokine production and upregulation of the NF-kB pathway. However, the absence of Stat3 in the supporting cells resulted in reduced microtubule stability. In conclusion, Stat3 is a critical protein for the sensory epithelium of the cochlea and hearing and functions in a cell and function-specific manner.

## 1 Introduction

The organ of Corti is the receptor organ for hearing located in the cochlea. It consists of various highly differentiated epithelial cells. The sensory signals of outer and inner hair cells are converted into action potentials, which are then transmitted to the auditory brainstem for further processing. Pillar and Deiters cells support the sensory hair cells basally and ensure stability during deflection of the basilar membrane due to endolymph fluid motion. The outer hair cells (OHC) amplify oscillations through fast mechanical contraction or elongation (Brownell et al., 1985), which is provided by the motor protein prestin (Zheng et al., 2000). Therefore, a high level of cytoskeletal structure and signaling pathway is required. The supporting cells consist of tightly bundled cytoskeletal microtubule arrays crosslinked with actin filaments to withstand shearing forces of auditory processing (Sugawara et al., 2004; Zetes et al., 2012).

In the cochlea, Stat3 is highly expressed in sensory hair cells and Deiters cells of the organ of Corti (Wilson et al., 2014) and the spiral ganglia Stat3 is a member of the family of “Signal Transducers and Activators of Transcription,” known for its DNA binding activity achieved through phosphorylation of Tyrosine 705 (Y705) via canonical pathway. This activated form is catalyzed via IL-6 transducing receptor chain gp130 and Jak2 (Boulton et al., 1995). In addition, unphosphorylated Stat3 (U-Stat3) can also act as a transcription factor by binding to importin α3 and Nf-κB (Liu et al., 2005; Yang and Stark, 2008). Additionally, Stat3 can be phosphorylated at Serine 7272 (S727). It is known that phosphorylated S727 of Stat3 (pStat3) regulates mitochondrial metabolism through the interaction of various mitochondrial proteins and gene expression via a non-canonical pathway (Macias et al., 2014; Wegrzyn et al., 2009). For translocation into mitochondria, pS727 Stat3 acts as a monomeric protein with GRIM19 and TOM20 (Tammineni et al., 2013). It is hypothesised to enhance electron respiratory chain activity, calcium release, ATP production and regulation of ROS by interacting with complexes I and II (Gough et al., 2013; Meier et al., 2017). Stat3 plays various important cellular roles and undergoes modifications depending on its pathway (Diallo and Herrera, 2022). Depending on the cell type and function, Stat3 is involved in cellular processes including proliferation, cell survival (Levy and Darnell, 2002; Levy and Lee, 2002), but also inflammation (Yu et al., 2009), autophagy (You et al., 2015), apoptosis (Wan et al., 2010) and microtubule dynamics (Ng et al., 2006), including cell growth. In immature organ of Corti, it could be shown that Stat3 signaling regulates cell division and regulates hair cell differentiation (Chen et al., 2017). However, little is known about the function of Stat3 in the sensory epithelia of the mature cochlear duct and its role in hearing. To evaluate the role of Stat3 in the organ of Corti, the inducible Cre/loxp system was used. An OHC cell-specific prestin-Cre mouse was selected that has a Cre expression pattern that more closely mirrors the endogenous expression of prestin. In addition, an Fgfr3 promoter-driven transgenic mouse line was chosen for Fgfr3-expressing pillar and Deiters cells in the mature cochlea (Hayashi et al., 2010). This study shows that Stat3 acts in a cell-and function-specific manner in the different cells of the cochlear sensory epithelium, thereby ensuring proper function of the organ of Corti.

## 2 Materials and methods

### 2.1 Animals

Slc26a5-iCre (B6J;129SV-Tg(Slc26a5^tm4-cre/ERT2^)^Jnz^, MGI:5316514) (Fang et al., 2012) or Fgfr3-iCre (B6;CBA-Tg(Fgfr3-iCre/ER^T2^)^4-2Wdr^, Jax:025809) (Young et al., 2010) were crossed with Stat3-fl mice (B6J;Cg(Stat3^tm2^)^Aki^; MGI:1926816) (Takeda et al., 1998). For activation of recombinase, the offspring received a daily intraperitoneal injection of tamoxifen (75 mg/kg body weight) between postnatal days (p)16 and 19 and either to generate Stat3^ΔOHC^ in the outer hair cells (Slc26a5-iCre::Stat3-fl) or Stat3^ΔSC^ in Deiters and pillar cells (Cox et al., 2012) (Fgfr3-iCre::Stat3-fl). The cell specific activity and inducibility of Cre was evaluate by cross breeding with a reporter line expressing tdTomato (B6.Cg-Gt(ROSA)26Sor^tm14(Cag-tdTomato)Hze/J^, Jax:007914). The experimental procedures were performed according to German regulations on animal welfare in agreement with and under control of the local veterinary authority and Committee on the Ethics of Animal Experiments (license number 55.2-DMS-2532-2-686-15).

Animals were housed under controlled conditions with a temperature range of 20–22°C, humidity between 55–65%, and a 12:12 h light/dark cycle. Food and water were provided *ad libitum*, and animal vitality and health were monitored daily. Audiometric testing was performed on Stat3^ΔOHC^ (*n* = 6) or Stat3^ΔSC^ (*n* = 6) and their untreated littermates (control) (*n* = 6) on p35. To minimize confounding effects on the results, all mice used for audiometry were carefully matched the same amount for each sex, while no gender-related differences were found. For anesthesia, a combination of ketamine hydrochloride (75 mg/kg body weight, Ketavet 100, Pharmacia) and xylazine hydrochloride (5 mg/kg body weight, Rompun 290, Bayer) was injected intraperitoneally at a volume of 1 mL/kg body weight. Anesthesia was maintained by administering 10% of the initial dose at 30-min intervals. Body temperature was regulated at 37°C with a temperature-controlled heating pad.

### 2.2 Audiometric assessment

Hearing function assessment was performed using the Tucker-Davis Technologies Inc. (TDT) setup, which included the SigGenRZ software and the TDT BioSigRZ system. The equipment used for loudspeaker control, microphone (378C01, PCB Pieztronics Inc., NY, United States), acquisition, processing, averaging, and data management was coordinated using the RZ6 Multi I/O Processor System. Auditory tests were performed as previously described (Bieniussa et al., 2022).

### 2.3 mRNA bulk sequencing

The mRNA of Stat3^ΔOHC^ (*n* = 3) and control (*n* = 4) was sequenced at the Core Unit SysMed of the University Hospital of Würzburg. To achieve this, the organ of Corti was carefully separated from the cochlea and digested with a lysis buffer containing 1 mg/mL Collagenase IV (Merck, #C4-28), 2 mg/mL Papain (Roth, #89331) and 1 mg/mL Thermolysin (Promega, #V4001) for 10 min at 37°C. Subsequently, OHCs were incubated with Slc26a5-antibody (rabbit, LS-C199978, LS. Bio.) and then with lgG anti-rabbit microbeads (Miltenyi Biotec, #130048602) for indirect selection of OHCs. The cell suspension was passed through MACS^®^ Separator columns (Miltenyi Biotec, # 130042401) to enrich magnetically labeled OHCs. After elution of OHCs, the RNA was extracted using the Arcturus Pico Pure Isolation Kit (Applied Biosystems, #15295033). The RNA samples were checked using 2,100 Bioanalyzer with RNA 6000 Pico kit (Agilent Technologies, #50671513) and DNase (Thermo Fisher, #EN0521) treatment was performed. cDNA libraries suitable for sequencing were prepared with SMARTer^®^ Stranded Total RNA-Seq Kit v3—Pico Input Mammalian (Takara, #634485, 634,486, 634,487, 634,488) according to manufacturer’s instructions. The PCR amplification was performed using 16 PCR cycles. Libraries were quantified by Qubit™ dsDNA HS Assay Kit (3.0 Fluometer; ThermoFisher, #Q32854) and quality was checked using 2,100 Bioanalyzer with High Sensitivity DNA kit (Agilent Technologies, #5067-4626) before equimolar pooling. Sequencing of pooled libraries was performed in paired-end mode with 75 nt read length on the NextSeq 500 platform (Illumina). Demultiplexed FASTQ files were generated with bcl-convert v.4.0.3 (Illumina). Gene expression was considered downregulated when there was a log2fold exchange of-1 with a significance of *p* < 0.05. Conversely, gene expression was considered upregulated when there was a log2fold exchange of 1 with a significance of p < 0.05, compared to control mice. Identified genes were analysed by Metascape to enrich involved pathways (Zhou et al., 2019).

### 2.4 Immunohistochemistry

For immunohistochemical analysis, mice were deeply anesthetized with CO_2_ and perfused intracardially with 4% PFA (Roth, #03353) in 1 M PBS pH 7.4 at room temperature. Cochleae were postfixed over night with 4% PFA in 1 M PBS pH 7.4 and afterwards decalcified in 125 mM EDTA (Invitrogen, #AM9262) for 24 h. For cryosections, the cochleae were incubated in ascending concentrations of sucrose (Roth, #90971) along with an additional suspension of Compound Tissue Tek (Sakura, #4583). The cochleae were then embedded in pure Compound Tissue Tek and sectioned at 9 μm. For whole mount staining, the cochlear turns were separated and covered in a 24 well plate with 1%PFA in 1 M PBS till preparations are finished. Afterwards the tissue was blocked and permeabilized with a solution of 10% normal horse serum (Merck, #H0146), 1% bovine serum albumin (Roth, #80762), 1% Triton X-100 (Serva, #37240), and 0.1% Tween20 (Sigma, #P1379) in 1 M PBS pH 7.4. The cochlear tissue was then incubated overnight on a 3D rotator with a primary antibody solution containing 3% normal horse serum, 1% bovine serum albumin, 0.3% Triton X-100, and 0.1% Tween20 with primary antibodies. After washing steps, secondary fluorochrome-conjugated antibodies were added according to manufacturer’s instructions for indirect labeling. Used antibodies and dilution can be seen in Supplementary Table S1.

### 2.5 Data analysis

The values of the DPOAE measurements were converted before analysis, as previously described (Bieniussa et al., 2022). Auditory brainstem response thresholds were defined at the last reproducible waveform of the recording. Values from all animals in each group were averaged and presented as hearing thresholds ± standard errors of the mean. Analysis of Stat3 expression in Deiter’s cells were performed with immunostaining and ImageJ ROI manager. The mean grey value is defined as the average of the values of all pixels, divided by the number of pixels within the selected area. The grey scale range extends from 0 (black) to 4,095 (white), with the latter value representing a saturated output. For the purposes of visualization, colors were set virtually. Statistical analysis and graphing were performed using GraphPad Prism software (GraphPad, San Diego, CA, United States). A *p*-value less than 0.05 was considered as significant. Significance was determined by comparison with control littermates. Image processing was performed with ImageJ and Photoshop CS9 (Adobe).

## 3 Results

### 3.1 Stat3 expression is absent in Stat3^ΔOHC^ and Stat3^ΔSC^, but exhibit normal morphology of the organ of Corti

**Figure 1:**
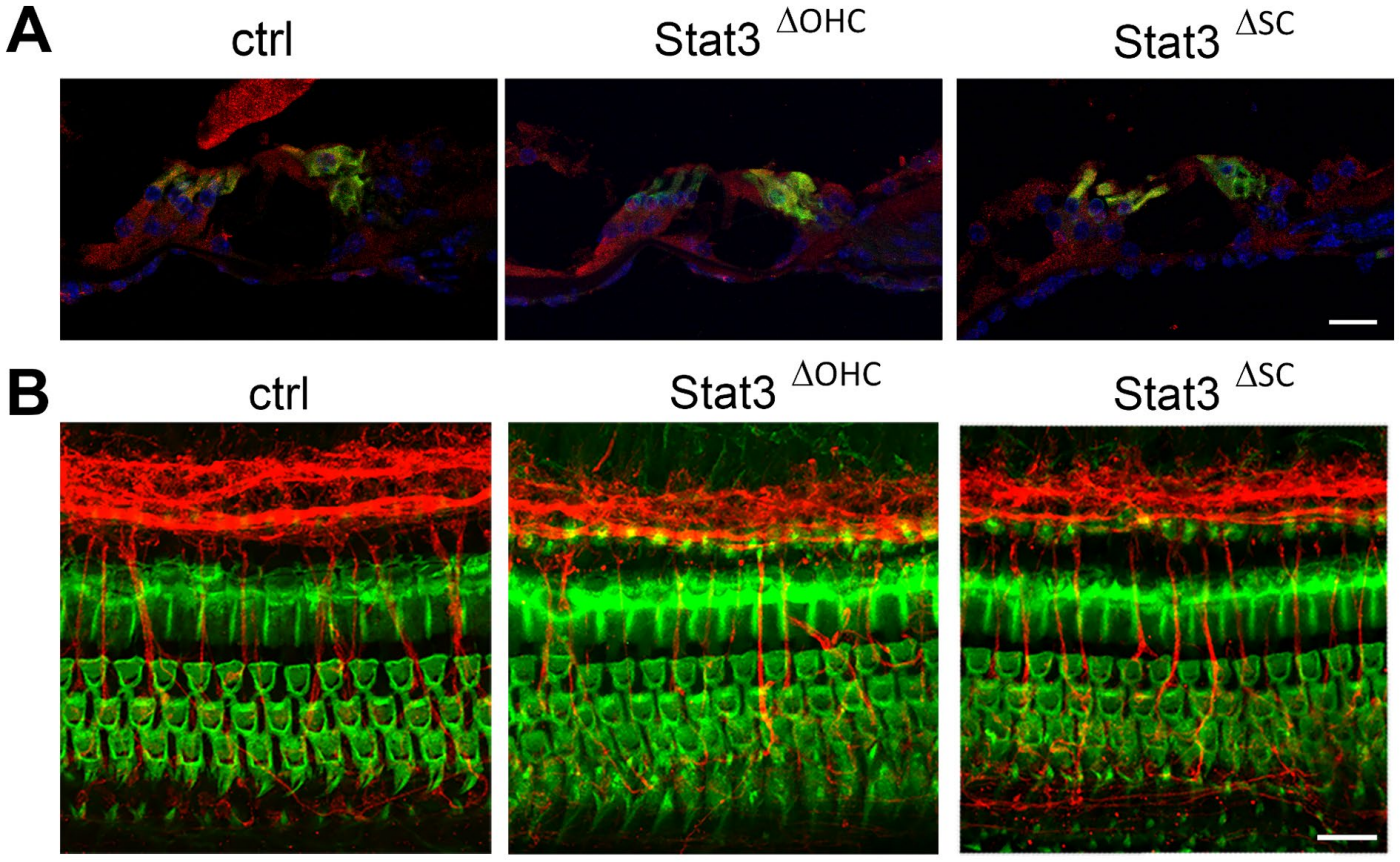
Immunohistochemical analyzation of Stat3^ΔOHC^ and Stat3^ΔSC^. **(A)** Stat3 (red) is highly expressed in sensory hair cells (green, Myo7a) or supporting cells in control cochleae, but is reduced in OHC in Stat3^ΔOHC^ and in supporting cells of the Stat3^ΔSC^ mice cochleae. **(B)** However, immunohistochemical analyzation of the cochlear duct indicated no cell loss of OHC (green, Phalloidin), altered innervation (red, βIII-tubulin) or morphology of the sensory epithelium of the cochlea. Ctrl: *n* = 3, Stat3^ΔSC^: *n* = 3. Scale bars: 20 μm.

Immunohistochemical evaluation of cryosections revealed that Stat3 is highly expressed in sensory hair cells and Deiters cells in the organ of Corti, as described in Wilson et al. (2014). Using CAG-tdTomato reporter line, Cre activity of Slc26a5 and Fgfr3 was detected. While Slc26a5 was outer hair cells specific (Supplementary Figure S1), tdTomato in Fgfr3-iCre was detected in Deiter cells, pillar cells and a small amount of cells in the spiral lamina and stria vascularis (Supplementary Figure S1). However, Stat3 expression is reduced in OHCs in Stat3^ΔOHC^ or in supporting cells in Stat3^ΔSC^ compared to control organ of Corti (Figure 1A). Notably, these images indicated no loss of OHCs or supporting cells in the sensory epithelium of the cochlea after conditional knock out of Stat3. Whole mount staining of Stat3^ΔOHC^ and Stat3^ΔSC^ cochleae revealed a normal morphology of the cochlear duct (Figure 1B). The results suggest that there is a reduced expression of Stat3 protein in OHCs in Stat3^ΔOHC^ and supporting cells in Stat3^ΔSC^. However, there was no cell loss observed in the sensory epithelium of the cochlea.

### 3.2 Stat3^ΔOHC^ and Stat3^ΔSC^ mice have increased hearing thresholds

**Figure 2:**
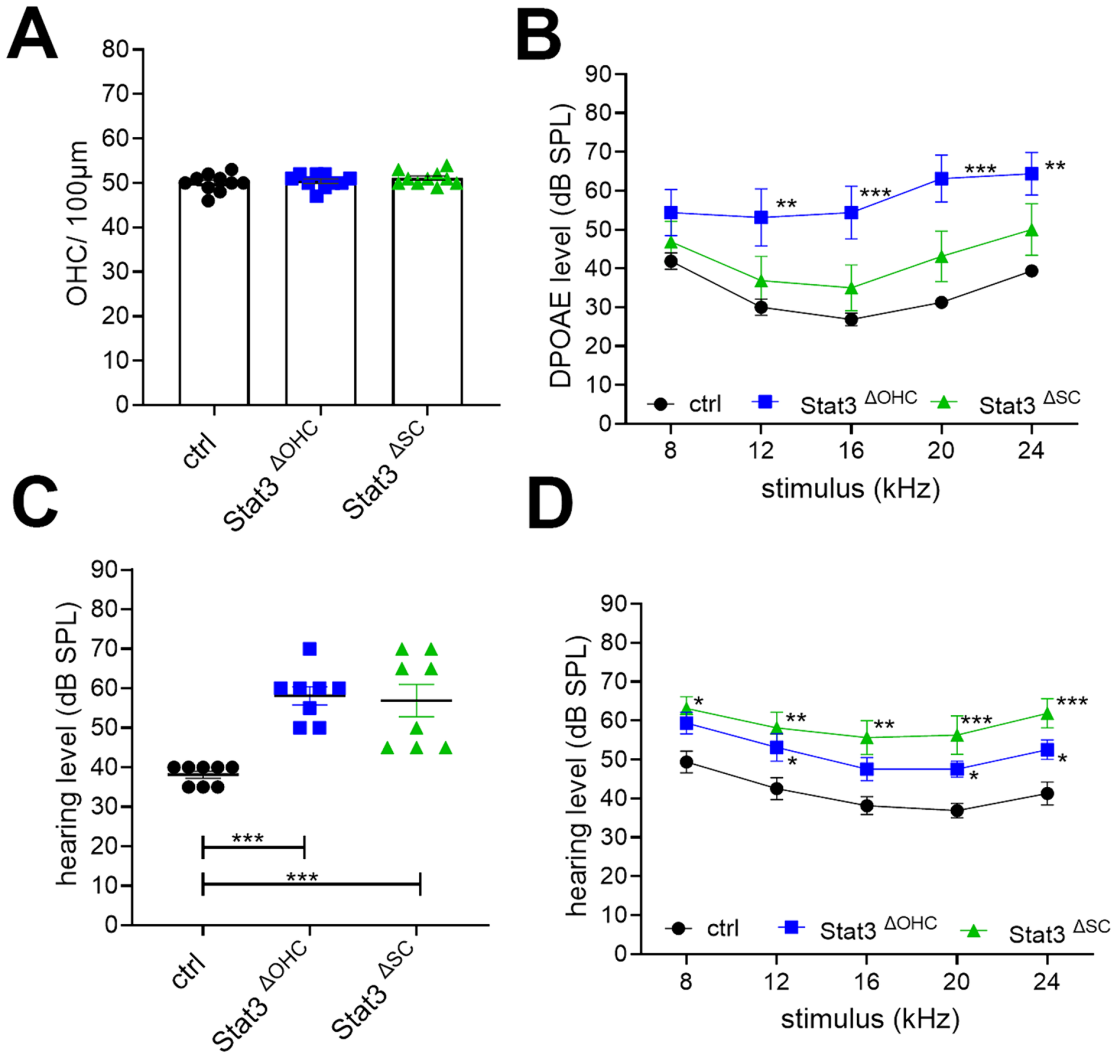
Audiological evaluation of Stat3^ΔOHC^ and Stat3^ΔSC^. **(A)** The number of OHCs was not altered, however **(B)** DPOAE levels were significantly reduced at 12 (*p* < 0.01), 16, 20 and 24 kHz (*p* < 0.001) of Stat3^ΔOHC^ (*n* = 6) but not of Stat3^ΔSC^ (*n* = 6). **(C)** Click ABR showed an increased hearing level of Stat3^ΔOHC^ and Stat3^ΔSC^ up to 20 dB SPL compared to control animals (*p* < 0.001). **(D)** In addition, tone ABR performance revealed a significantly reduced hearing level at 12, 20 and 24 kHz (*p* < 0.05) of Stat3^ΔOHC^ and at 8 (*p* < 0.05), 12, 16, 20 and 24 kHz (*p* < 0.001) of Stat3^ΔSC^. Ctrl, Stat3^ΔOHC^, Stat3^ΔSC^: *n* = 6. DPOAE and Tone ABR: Ordinary two-way ANOVA with Dunnett’s multiple comparison test. Click ABR: One-way ANOVA with Dunnett’s multiple comparison test. Significances: *p < 0.05, **p < 0.01, ***p < 0.001.

In the next step, the audiological electrophysiology was carried out by using the small animal audiometry technique. DPOAE thresholds at 12 (*p* < 0.01), 16, 20 (*p* < 0.001), and 24 kHz (*p* < 0.01) were significantly increased at Stat3^ΔOHC^ animals compared to ctrl littermates, but DPOAE measurements of Stat3^ΔSC^ were not significantly altered (Figure 2A). Nevertheless, the amount of OHCs per 100 μm of Stat3^ΔOHC^ (50.35 ± 0.57) and Stat3^ΔSC^ (50.14 ± 0.5) were not altered compared to control cochleae (51.48 ± 0.64) (Figure 2B). In addition, click-ABR showed a significant increase in hearing level for Stat3^ΔOHC^ with 59.58 ± 1.89 dB SPL and Stat3^ΔSC^ with 55.91 ± 3.29 dB SPL compared to control littermates with 36.67 ± 1.1 dB SPL (Figure 2C). In addition, tone-ABR levels were significantly increased at 8 (*p* < 0.05), 12, 16 (p < 0.01), 20, and 24 kHz (p < 0.001) in Stat3^ΔOHC^ mice and at 8, 20, and 24 kHz (p < 0.05) in Stat3^ΔSC^ (Figure 2D). The results showed that a loss of Stat3 in OHCs leads to a decreased activity of OHCs with decreased hearing level, but no loss of OHCs. However, deletion of Stat3 in Deiter’s and pillar cells in the organ of Corti resulted in increased hearing thresholds compared to control littermates. Taken together, these results indicate that Stat3 plays an important role in the sensory epithelial cells of the organ of Corti for correct hearing.

### 3.3 Outer hair cells of Stat3^ΔOHC^ showed an increase in inflammatory transcripts

**Figure 3:**
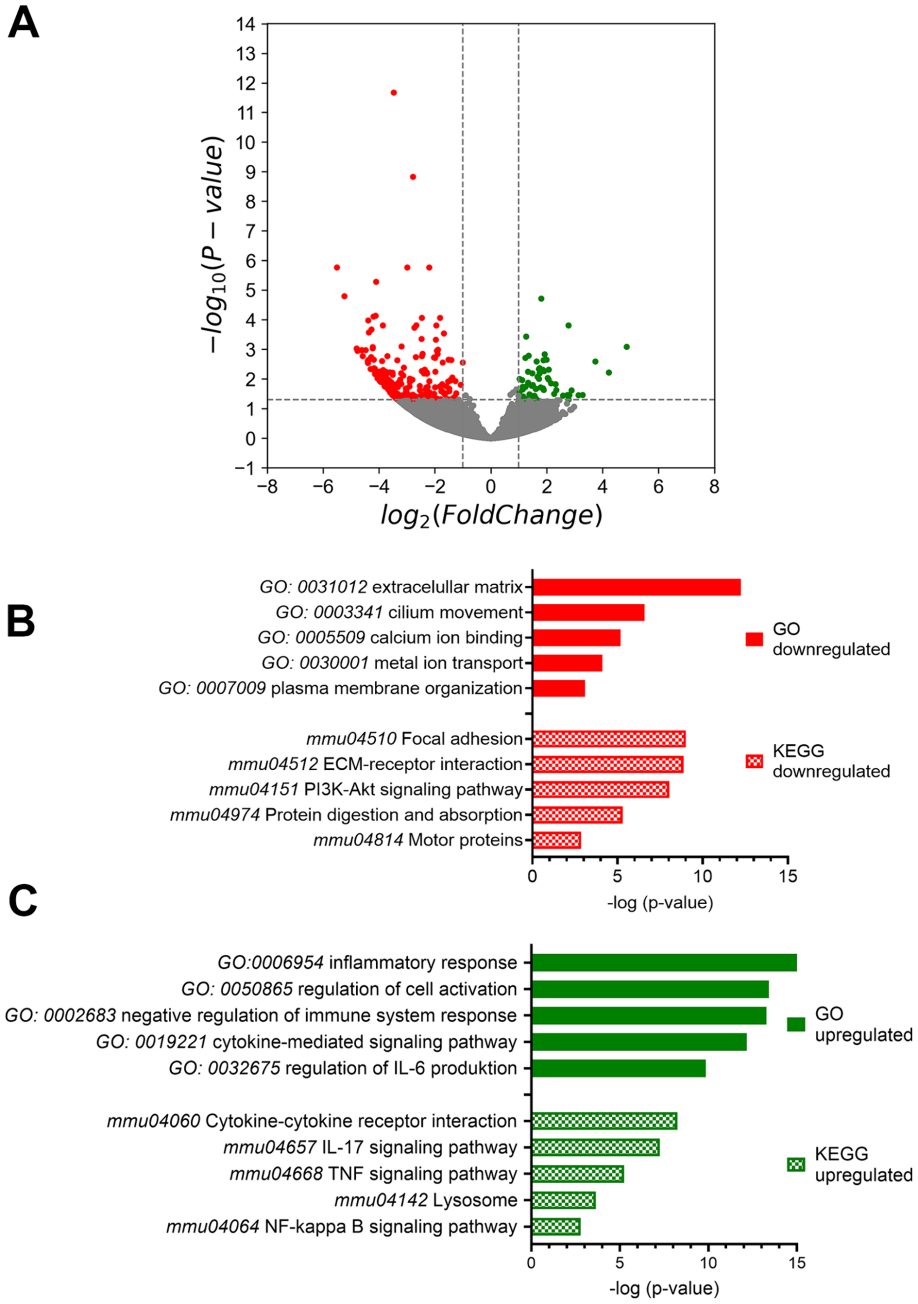
Bulk mRNA sequencing analysis. **(A)** The mRNA expression volcano plot indicates downregulation when there was a log2fold change of -1 with a significance of *p* < 0.05. Conversely, upregulation was indicated when there was a log2fold change of 1 with a significance of *p* < 0.05 compared to control mice. **(B)** Gene Ontology (GO) and Kyoto Encyclopedia of genes and Genomes (KEGG) term analysis of down- and **(C)** up-regulated genes performed by Metascape. Ctrl: *n* = 4, Stat3^ΔOHC^: *n* = 3.

Since pY705-Stat3 as well as U-Stat3 act as transcription factors, the cells were evaluated by bulk sequencing analysis to identify changes to regulatory pathways. Sequencing results revealed 476 downregulated genes and 58 upregulated genes (Figure 3A). To further investigate potentially altered pathways, we performed Gene Ontology (GO) and Kyoto Encyclopedia of Genes and Genomes (KEGG) term analysis using Metascape databank and graphed after –log (*p*-value). The most downregulated transcripts after using GO encode database, were involved in extracellular matrix (12.234), cilium movement (6.577), calcium binding (5.168) and metal ion transport (4.09) as well as plasma membrane organization (3.085) (Figure 3B). Metal ion transport included also potassium ion transport, the transmembrane transport and potassium channel activity (Supplementary Table S2). KEGG analysis of downregulated genes matches in pathways of focal adhesion (9.071), ECM receptor interaction (8.865), PI3Akt signaling pathway (8.042), protein digestion (5.298), which are part of the extracellular matrix pathway and motor protein pathways (2.865), which are connected to the cilium movement (Figure 3B; Supplementary Table S2). On the other hand, the upregulated transcripts can be associated with inflammatory response (16.268), regulation of cell activation (13.43), negative regulation of immune system response (13.281) but activation of cytokine mediated signaling pathway (12.172) and a positive regulation of IL-6 production (9.86) (Figure 3C; Supplementary Table S2). KEGG database popular matches of upregulated genes included IL-17 signaling pathway (6.696) with TNF signaling pathway (4.828), which belongs to regulation of cell activation, cytokine-cytokine receptor interaction (5.891) as well as NF κB signaling (2.784), which come under the cytokine mediated signaling pathway (Figure 3C; Supplementary Table S2).

### 3.4 Supporting cells of Stat3^ΔSC^ indicated a instability of phalangeal processes

OHCs are important for the amplification of sound and has a high rate of transcription. In contrast, supporting cells are highly differentiated cells with a stable cytoskeleton of actin and microtubule bundles and are important for the stability of the organ of Corti. To investigate this further, we analyzed the cytoskeleton of supporting cells in the mature cochlea by immunohistochemical study of stable posttranslational modification of microtubules.

**Figure 4:**
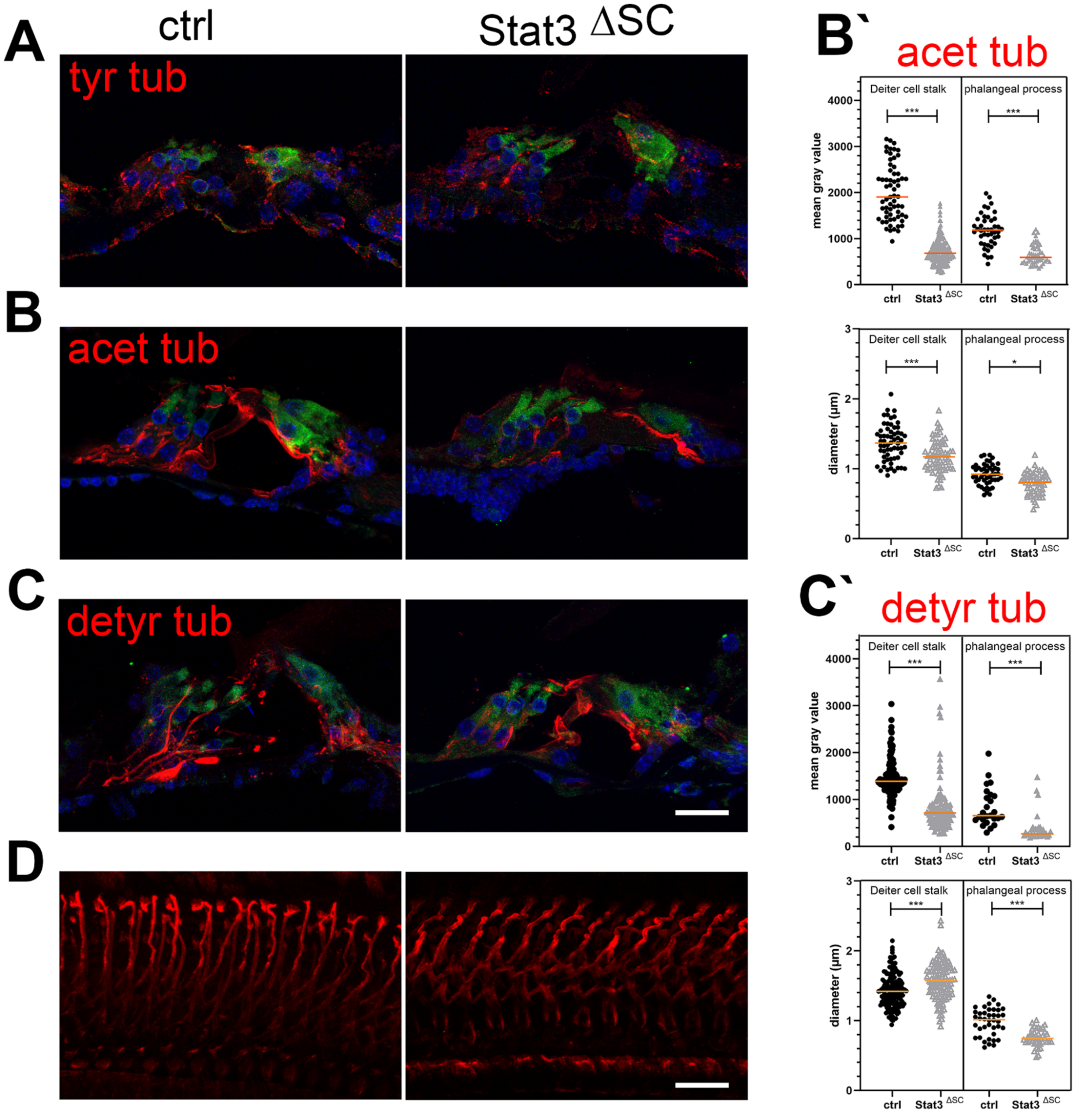
Immunohistological analysis of post-translational modifications of microtubules in Stat3^ΔSC^ organ of Corti cryosections with OHC marker Myosin 7a (green). **(A)** Tyrosinated tubulin (tyr tub, red) is expressed on sensory hair cells, nerve fibers and supporting cells of the sensory epithelium of the cochlea. **(B)** Acetylated tubulin (acetyl tub, red) is expressed in Deiters and pillar cells, but are significantly reduced in Stat3^ΔSC^. **(B′)** Statistical analyzation of acetylated tubulin in mean gray value and the diameter of Deiters cells stalk base and phalangeal processes showed decreased acetylated tubulin (*p* < 0.001) and reduced diameter in the stalk base (*p* < 0.001) and phalangeal processes (*p* < 0.05) inStat3^ΔSC^. **(C)** Detyrosinated tubulin (detyr tub, red) is expressed in Deiters and pillar cells, but are significantly reduced in Stat3^ΔSC^. **(C′)** Statistical analyzation of detyrosinated tubulin in mean gray value of Deiters cells stalk base and phalangeal processes showed decreased acetylated tubulin (*p* < 0.001). However, diameter of Deiters cells stalk of Stat3^ΔSC^ is significantly higher compared to control Deiters cells (*p* < 0.001), whereas the phalangeal processes of Stat3^ΔSC^ is significantly reduced compared to control phalangeal processes. **(D)** Whole Mount staining with detyrosinated tubulin revealed well-defined structures of detyrosinated microtubule bundles in control cochlea, whereas these modified bundles show progressive instability with high tortuosity. Statistical analyses: Unpaired *t*-test, two-tailed. Ctrl: *n* = 3, Stat3^ΔSC^: *n* = 3. Scale bars: 20 μm. Significances: *p < 0.05, ***p < 0.001.

In cryosections, dynamically unstable tyrosinated tubulin was found in cochlear sensory hair cells, nerve fibers, and supporting cells in both control mice and Stat3^ΔSC^ (Figure 4A). Acetylated and detyrosinated tubulin was found predominantly in the stalk and phalangeal processes of Deiters cells in control animals (Figures 4B,C). Acetylated tubulin in the stalk (1,998 ± 76.07) and in the phalangeal processes (1,178 ± 52.61) of control Deiters cells is significantly higher (*p* < 0.001) compared to stalk base (718.4 ± 21.87) and phalangeal process (660.5 ± 29.3) of Stat3^ΔSC^ Deiters cells (Figures 4B,B′). Additionally, the diameter of acetylated tubulin in Stat3^ΔSC^ Deiters cells stalk base with 1.18 ± 0.03 μm (*p* < 0.001) and phalangeal process 0.79 ± 0.02 μm (*p* < 0.05) is significantly reduced compared to control Deiters cells structures (stalk: 1.37 ± 0.03 μm, phalangeal process: 1 ± 0.09 μm) (Figure 4B′).

For detyrosinated tubulin the mean gray value in Deiter cells stalk base (829.6 ± 50.81) and phalangeal processes (392 ± 53.25) of Stat3^ΔSC^ is significantly reduced (*p* < 0.001) compared to stalk (1,449 ± 30.66) and phalangeal processes (832.4 ± 81.19) of control Deiters cells (Figures 4C,C′). However, the diameter of stalks of Stat3^ΔSC^ are significantly increased with 1.57 ± 0.02 μm compared to the control Deiters cells 1.43 ± 0.02 μm (p < 0.001) (Figures 4C′,D), whereas the diameter of detyrosinated tubulin in phalangeal processes of Stat3^ΔSC^ Deiters cells (0.75 ± 0.01 μm) is significantly reduced compared to control structures (0.98 ± 0.03 μm, *p* < 0.001) (Figure 4C′). Moreover, immunostaining of detyrosinated microtubules bundles in Whole Mounts revealed that the microtubule bundles were less stable in shape and exhibited multiple curvatures compared to the supporting cells of control cochleae (Figure 4D). This finding demonstrates a clear difference in microtubule structure and highlights the importance of further investigation into the role of post-translational modification of microtubules and their dynamics in the supporting cells of the sensory epithelium of the cochlea and hearing performance.

## 4 Discussion

Stat3 plays a critical role in regulating various processes in a cell-specific manner. It is highly expressed in sensory hair cells and epithelial cells in the cochlea and is critical for embryonic cell development and noise-induced hearing loss (Chen et al., 2017; Wilson et al., 2014). Moreover, Stat3 is a transcription factor and is essential in the adult stage to balance cell survival and apoptosis, as well as microtubule dynamics (Levy and Darnell, 2002; Levy and Lee, 2002; Ng et al., 2006). This study clearly demonstrate that Stat3 plays a crucial role both OHC activity and hearing performance in mature cochlea. Specifically, in supporting cells, Stat3 regulates microtubule dynamics and their modification rate. Any defects in these dynamics can lead to instability of the organ of Corti, resulting in hearing impairment. Altogether, it can be concluded that the function of Stat3 in the organ of Corti is cell specific.

### 4.1 Absence of Stat3 in OHC and SC caused hearing impairment

The absence of Stat3 in OHCs in the mature cochlea leads to an increase in DPOAE level and overall hearing thresholds compared to control controls. However, no cell loss was found due to the absence of Stat3. DFNB59 knockout mouse (Delmaghani et al., 2006) has revealed that sensory hair cell defects alter electrophysiological properties. Pejvakin, a protein of unknown function in sensory hair cells and spiral ganglia, is the cause of DFNB59 due to a mutation. With this evidence, we can confidently conclude that DFNB59 has a significant impact on the electrophysiological properties of sensory hair cells. It is interesting to note that both mutant mice and affected patients show auditory brainstem responses (ABRs) (Delmaghani et al., 2006; Delmaghani et al., 2015) with absence of distortion product otoacoustic emissions (DPOAEs) but no early OHC loss (Schwander et al., 2007). The results of our experiments were found to be consistent in terms of both audiological and histological outcomes. Despite the absence of any detectable loss of outer hair cells, the DPOAE thresholds are elevated. These results may be interpreted as evidence of an inability of the OHCs to process sensory information. Pejvakin mutants have been shown to result in increased oxidative stress, characterized by the overproduction of ROS (Delmaghani et al., 2015). Stat3 can be phosphorylation at S727, which enables it to interact with mitochondrial metabolism and regulate ROS production. It may therefore be hypothesized that Stat3 deficiency could influence ROS production in OHCs. Consequently, further investigations are required to evaluate the role of Stat3 in relation to mitochondria metabolism and regulation in OHCs.

However, not only OHCs are important for adequate hearing function, but also the supporting cells surrounding the OHCs play a critical role. Our experiments have shown that the absence of Stat3 in Fgfr3-expressing supporting cells of the organ of Corti of mice leads to an increase of the hearing thresholds in the click and tone ABR. In the cochlea of guinea pig, Fgfr3 can be detected in spiral ganglion neurons, sensory hair cells of the cochlea as well as supporting cells like pillar, Deiter and Hensen’s cells (David et al., 2002). Nevertheless, previous study could shown that the Fgfr-iCre mice shows different expression pattern in the supporting cells of the organ of Corti depending on recombinase activation. In the mature cochlea, only pillar and Deiter’s cells were effected by tamoxifen induction (Cox et al., 2012) (Supplementary Figure S1), confirming Fgfr3 expression in the mature cochlea (Hayashi et al., 2010). This clearly demonstrates the importance of these supporting cells in the organ of Corti for maintaining good hearing. We can confidently say that our findings have significant implications for the field of audiology. Previous studies have shown that mechanical properties and anatomical arrangement play a crucial role in determining the relative motion of the cochlear duct. Deiter cells, due to their stiffness, act as a mechanical microequalizer for OHCs, making them incredibly important for cochlear response and recovery (Lukashkina et al., 2022; Zhou et al., 2022). In addition, Xia et al. (2022) have shown that a reduction in cell arrangement leads to hearing loss. These data suggest that disruption of the architecture of supporting cells in the mature cochlea is a critical factor in maintaining proper hearing. This discovery changes the way we think about hearing loss and the supporting cells of the sensory epithelium of the cochlea.

### 4.2 Stat3 suppresses cytokine-mediated metabolism in OHC

Stat3 is a crucial transcription factor for multiple proteins and that plays a vital role in microtubule dynamics and DNA transcription factor function. Its activity is regulated by phosphorylation of two separate residues, Y705 and S727, which are essential for its diverse function (Cheng et al., 2017; Mohr et al., 2012; Wegrzyn et al., 2009; You et al., 2015). Upon phosphorylation of its tyrosine 705 (pY705) by Janus kinases, Stat3 homodimerizes, enters the nucleus, binds to Stat3 response elements, and triggers transcription of its target genes. Not only phosphorylated Stat3, but also unphosphorylated Stat3 plays a significant role in cells. U-Stat3 acts as a transcription factor and enhances the expression of various genes, after binding to importin α3 and NF-κB. Interestingly, NF-κB signaling pathway was upregulated after gene enrichment (Supplementary Table S2). U-Stat3 leads to a late response to IL-6 stimulation (Yang and Stark, 2008; Yang et al., 2007), which can also be seen in the upregulation IL-6 production (Figure 3; Supplementary Table S2). These findings highlight the crucial role of U-Stat3 in cellular processes. The results demonstrate that Stat3 act as a transcription factor in the OHCs and an absence of Stat3 leads to a late response via IL6 production and thereby upregulate inflammatory pathways.

OHCs act as amplifiers by altering their length through a motor protein called Prestin. This unique movement is triggered by depolarization, achieved by a deflection of the stereocilia and the opening of the mechanoelectrical transduction (MET) channels (Caprara and Peng, 2022). Through open MET channel and signaling pathway cascade, cations are depolarized in the OHCs, while in the end a rescue of the endocochlear potential is initiated. These signaling pathways are crucial for maintaining hair cell physiology. In the mutant OHCs, calcium ion binding signaling as well as potassium ion transport and channel activity is downregulated due to the absence of Stat3 (Supplementary Table S2). In OHCs, it is known that a disturbance of potassium fluxes caused hearing loss (Ruttiger et al., 2004). Moreover, a calcium imbalance leads to vulnerability in outer hair cells especially in high frequency range (Fettiplace and Nam, 2019). In summary, a disturbed cation homeostasis in OHC leads to a dysfunction of temporal precision of sound processing (Ceriani and Mammano, 2012; Patuzzi, 2011; Peixoto Pinheiro et al., 2021). While this study just indicated a gene ID evaluation, further investigations are required to underline cation disturbances in OHCs of these mice.

### 4.3 Stat3 regulates microtubule dynamics in supporting cells

While the OHCs amplify the detected sound, the supporting cells play a crucial role in passively withstanding these forces due to their precise arrangement, which provides a strong structural scaffold for the sensory cells on the sensory epithelium. This remarkable design of the cochlea allows incredible auditory capabilities. To withstand the continued force in the cochlear duct, supporting cells possess a stable cytoskeletal structure of actin and microtubule bundles for a high stiffness. Posttranslational modifications of microtubules affect their stability and influencing the polymerization and depolymerization dynamic (Aillaud et al., 2017; Bahi-Buisson and Maillard, 1993). Tyrosinated tubulin supports depolymerization, while detyrosination maintains polymerization (Infante et al., 2000; Peris et al., 2009), which we were able to identify in Deiter cells stalks of Stat3^ΔSC^ (Figure 4B′). A decrease rate of detyrosinated tubulin can cause an imbalance in microtubule dynamics and a higher rate of microtubule depolymerization (Peris et al., 2009). The effect of changes in the expression of stable tubulin acetylated and detyrosinated on the stability of supporting cells can be investigated using atomic force microscopy, as has been done in another microtubule deficient model of the organ of Corti (Chen et al., 2021). These results support the hypothesis that supporting cell stiffness is necessary for proper cochlear micromechanics and that an unstable architecture of supporting cells leads to dysfunction of outer hair cells and hearing loss.

In addition, Stat3 plays an important role in counteracting the effects of phosphorylated Stathmin1, a key player in microtubule depolymerization (Moreno and Avila, 1998; Verma et al., 2009). They regulate both the growth and disassembly phases of microtubules. Consequently, the absence of Stat3 could promote tubulin depolymerization via Stathmin1, leading to cytoskeletal instability in supporting cells. Previous studies have shown that Stathmin1 deficiency can cause a disruption of microtubule dynamics and defective MTOC polarization (Filbert et al., 2012; Silva and Cassimeris, 2013; Tian et al., 2012). On the other hand, Stathmin1 blockade, like Stat3 overexpression, has been discussed as a potential therapeutic issue to balance microtubule dynamics in neurodegenerative diseases (Gagliardi et al., 2022; Yadav et al., 2016). Therefore, further investigations are needed to analyze the role of Stathmin1 and Stat3 in microtubule dynamics in the supporting cell and this will help shed light on their potential contribution to the field.

### 4.4 Limitation

The Cre/loxp system bypasses the conventional knockout mouse model in which the deletion of a gene of interest is manifested in the germline and therefore in each cell of an organism. These models can be used to identify the early functions of a gene in the context of embryonic development, where a high proportion of early embryonic lethality is caused, as in Stat3^−/−^ mice (Takeda et al., 1997). The expression of Stat3 in the cochlea fluctuates during the embryonic and early postnatal period, but remains a high level at hearing onset (Chen et al., 2017). In the present study, the Cre/loxP system enables cell-specific and temporal dependent deletion of the Stat3 gene after hearing onset to highlight the importance of Stat3 for a correct hearing performance. The controlled expression of Cre enzymes is achieved by tamoxifen, a synthetic anti-estrogen. Tamoxifen increases the channel activity of Ca^2+^ voltage-sensitive potassium channel (BK-channel) (Dick et al., 2001; Duncan, 2005), which can disturb the efferent feedback loop (Thompson et al., 2006). In studies with noise exposure, a depletion of estrogen or induction of tamoxifen can potentiate the damaging effect and a reduction of hearing performances (Pillai and Siegel, 2011; Stenberg et al., 2003; Thompson et al., 2006). In our study, mice without tamoxifen injection were used as a control group. Thus, it cannot be excluded that tamoxifen may have a negative effect on hearing performances of Stat3^ΔOHC^ and Stat3^ΔSC^ mice, even though our study design did not include noise exposure.

## 5 Conclusion

Stat3 has a broad spectrum of interactions that are most likely cell and function specific in cells of the organ of Corti. These results highlight the importance of Stat3 in maintaining proper auditory function, which plays a critical role in sensory and epithelial cells for correct sound transduction. Specifically, Stat3 in OHCs regulates membrane organization and intracellular signaling pathways, and its absence leads to cytokine metabolism and activation of inflammatory response signaling. However, the absence of Stat3 in supporting cells has led to instability of the organ of Corti due to altered post-translational modifications of microtubules. To gain a better understanding of the role of Stat3 in the sensory epithelial cells of the cochlea and the hearing process in the mature cochlea, further molecular biology experiments should be performed to analyze the function and processes of these specific cells.

## Data Availability

The data presented in the study are deposited in the NCBI´S Gene Expression Omnibus database, accession number GSE278228.
