# Supplementary material for: Inactivity of Stat3 in sensory and non-sensory cells of the mature cochlea
Source: Front Mol Neurosci. 2024 Oct 14;17:1455136. doi: 10.3389/fnmol.2024.1455136 (PMC11513353; doi:10.3389/fnmol.2024.1455136)
Supplement: Supplementary file 1 [file Data_Sheet_1.PDF]

Supplementary information

Inactivity of Stat3 in sensory and non-sensory cells of the mature cochlea

Bieniussa L[1], Stolte C[1], Arampatzi P[2], Engert J[1], Völker J[1], Hagen R[1], Hackenberg S[1], Rak K[1]*

[1] Department of Oto-Rhino-Laryngology, University Hospital, Würzburg, Germany

[2] Core Unit System Medicine, University Hospital, Würzburg, Germany

**Antibody, houst**	**company**	**Article number**	**concentration**
**Primary antibody**			
Acetylated tubulin, mouse	Sigma	T7451	1:2000
Detyronisated tubulin, rabbit	Millipore	AB3201	1:2000
Myosin7a, mouse	DSHB Iowa	1381	1:500
Myosin7a, rabbit	Proteus BioScience	256790	1:1000
Slc26a5 , rabbit	LS Bioscinece	LS-C199978	1:1000
Stat3α, rabbit	Cell Signaling	8768	1:500
Tyronisated tubulin, mouse	Sigma	SAB4200776	1:2000
**Secondary antibody**			
DAPI	Cell Signaling	4083	1:5000
Donkey anti mouse Alexa 488	ThermoScientific	A32766	1:1000
Donkey anti rabbit Alexa 488	ThermoScientific	A32790	1:1000
Donkey anti mouse Alexa 555	ThermoScientific	A32773	1:1000
Donkey anti rabbit Alexa 555	ThermoScientific	A32794	1:1000

Table S1: Used primary and secondary antibody.

Table S2: Sequencing analyzation and Gene Enrichment after Metascape.

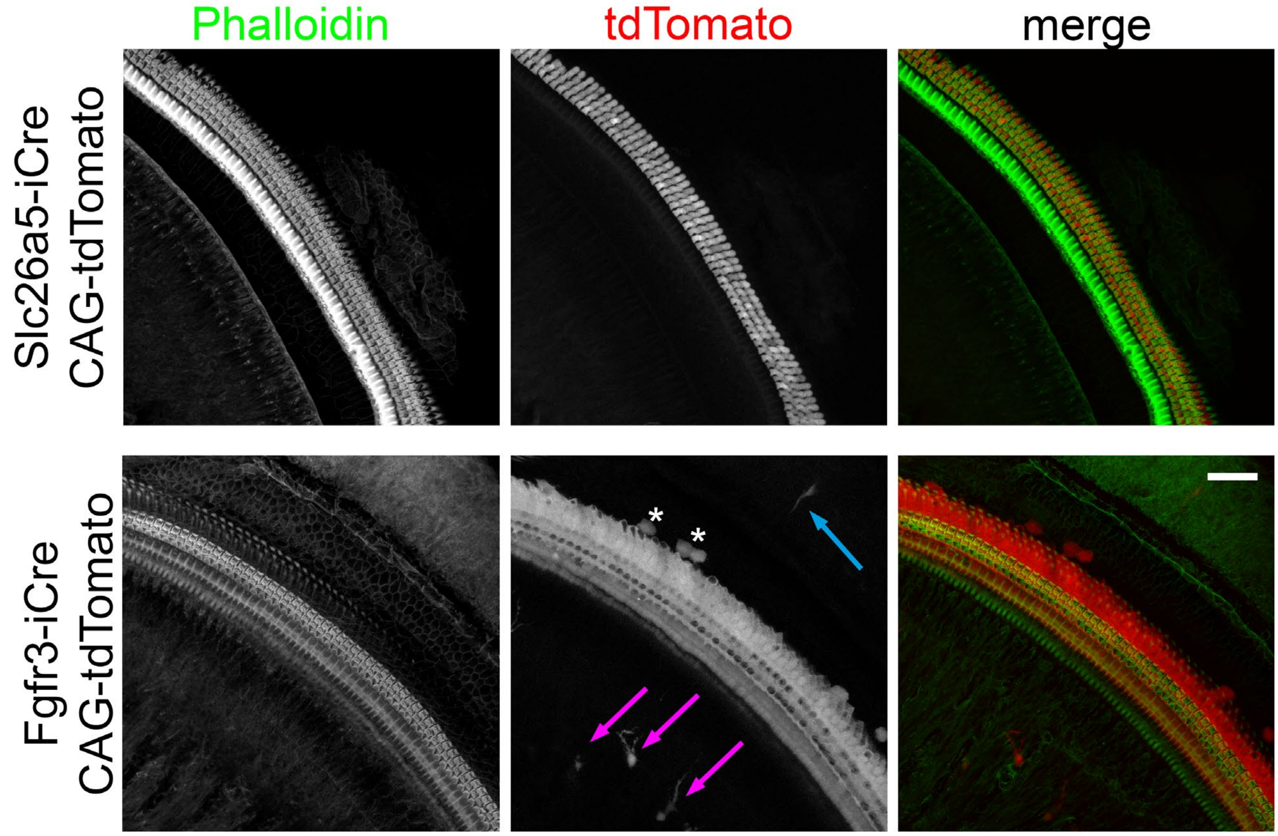

Figure S1: Whole mounts of Slc26a5-iCre and Fgfr3-iCre with reporterline CAG-tdTomato. In Whole Mount stainings of Slc26a5-iCre::CAG-tdTomato showed expression of tdTomato in all OHCs. Fgfr3-iCre::tdTomato indicated tdTomato expression in Deiters and pillar cells. Additionally, a few Claudius cells were labeled (white asterisk), glial cells in the spiral lamina (magenta arrow) and cells in the stria vascularis (blue arrow). Scale bar: 20 µm.
